# Corticosteroid Taper Duration and Relapse Rate in Immune Checkpoint Inhibitor-Induced Pneumonitis: A Meta-Analysis

**DOI:** 10.7759/cureus.103794

**Published:** 2026-02-17

**Authors:** Rodrigo Furlan Silva Fabri, Anvitha Soundararajan, Rodolfo Myronn De Melo Rodrigues

**Affiliations:** 1 Internal Medicine, Texas Tech University Health Sciences Center El Paso Paul L. Foster School of Medicine, El Paso, USA

**Keywords:** checkpoint inhibitor toxicity, corticosteroid tapering, immune-related pneumonitis, immunotherapy adverse events, relapse risk

## Abstract

Immune checkpoint inhibitor-induced pneumonitis is a serious immune-related adverse event managed primarily with systemic corticosteroids, yet the optimal taper duration needed to prevent relapse remains uncertain. High rates of recurrence during or shortly after taper completion continue to complicate clinical care. This systematic review and meta-analysis evaluate the pooled relapse rate following corticosteroid therapy and compare outcomes between fixed short-term tapers and individualized or longer taper strategies.

A systematic search of the published literature identified studies reporting quantifiable relapse events after corticosteroid taper for pneumonitis. Six cohorts from four studies, totaling 296 patients, met eligibility criteria. The pooled relapse proportion (estimated event rate) was calculated using a random-effects model with logit transformation. Comparative analysis between taper strategies was performed using a fixed-effects model, and sensitivity analyses were conducted to evaluate robustness.

The pooled relapse rate was 25.8%, indicating that approximately one in four patients experienced recurrence after corticosteroid treatment. Fixed six-week taper cohorts demonstrated significantly higher odds of relapse compared with variable or extended taper regimens. Sensitivity analysis, which excluded a low-event cohort, confirmed this association and reduced heterogeneity. Qualitative synthesis showed that certain clinical features, including the organizing pneumonia radiographic pattern and pneumonitis occurring after durvalumab therapy, frequently required tapers extending beyond twelve to seventeen weeks to achieve stable remission.

Approximately one quarter of patients treated for immune checkpoint inhibitor-induced pneumonitis experience relapse after corticosteroid therapy. Fixed short-term tapers are associated with significantly higher recurrence risk compared with individualized or longer taper strategies. These findings support a risk-stratified approach to taper duration, emphasizing the need for extended tapers in higher-risk clinical scenarios while monitoring for steroid-related adverse effects.

## Introduction and background

Immune checkpoint inhibitors have transformed treatment outcomes across multiple cancer types, yet their benefits are accompanied by a spectrum of immune-related adverse events. Immune checkpoint inhibitor-induced pneumonitis is one of the most clinically significant toxicities, with reported mortality rates ranging between 10 and 20% in severe presentations [1]. Although relatively uncommon compared with other immune-related adverse events, pneumonitis carries a disproportionate risk of morbidity and treatment disruption.

Systemic corticosteroids remain the standard first-line therapy for grade two or higher pneumonitis. Most contemporary guidelines recommend a taper duration of four to six weeks once clinical improvement has been achieved [2,3]. However, real-world experience increasingly suggests that relapse during or shortly after taper completion is frequent, with reported rates ranging from 15% to over 35%, and may lead to repeated episodes of respiratory decline, prolonged immunosuppression, rehospitalization, and interruption of anticancer therapy [4-7]. These clinical realities highlight the uncertainty surrounding the optimal duration of corticosteroid taper needed to prevent recurrence.

Retrospective studies have raised concerns that short, fixed tapers may be associated with higher relapse rates compared with individualized or extended tapering strategies [4-7]. Additional evidence indicates that patients with specific radiographic or clinical phenotypes, such as the organizing pneumonia (OP) pattern, or those receiving consolidation durvalumab after chemoradiation, may require substantially prolonged taper durations to avoid recurrence [5,7]. Real-world data further show that steroid-responsive and steroid-refractory pneumonitis represent distinct clinical trajectories, often necessitating different tapering strategies [8-12]. Chronic immune checkpoint inhibitor-induced pneumonitis, although less common, may require exceptionally long courses of corticosteroids, sometimes exceeding several months [9,10].

Despite these observations, taper duration remains highly variable, creating a clinical dilemma between the risk of pneumonitis relapse with shorter tapers and the cumulative toxicity of prolonged steroid exposure. This lack of evidence-based standardization hinders risk-stratified management. Moreover, the impact of corticosteroids on antitumor immunity and treatment efficacy remains an area of active investigation, adding further clinical uncertainty [10,13].

The purpose of this review and meta-analysis is to clarify the magnitude of relapse risk following corticosteroid treatment for immune checkpoint inhibitor-induced pneumonitis and to compare outcomes between fixed short-term tapers and individualized or longer taper regimens. By synthesizing quantitative and qualitative evidence, this study aims to identify patient and disease characteristics associated with higher relapse risk and to support a more rational, risk-stratified approach to taper duration in clinical practice.

## Review

Evidence search and study selection

This systematic review and meta-analysis were conducted in accordance with the Preferred Reporting Items for Systematic Reviews and Meta-Analyses (PRISMA) 2020 guidelines. A comprehensive literature search was performed using PubMed, PubMed Central, and major oncology journals from database inception through September 2025.

PubMed and PubMed Central were selected as primary databases because they capture the majority of peer-reviewed clinical studies on immune checkpoint inhibitor-induced pneumonitis and provide comprehensive indexing of oncology and pulmonary literature. Manual searches of major oncology journals were conducted to minimize the risk of missing eligible studies. Embase, Scopus, and Web of Science were not included due to feasibility constraints; this is acknowledged as a limitation. The search strategy included predefined databases, structured keyword combinations using Boolean operators, specified time limits, and eligibility criteria to support transparency and reproducibility.

The search strategy included combinations of the following keywords and Medical Subject Headings (MeSH): “immune checkpoint inhibitor pneumonitis,” “immune-related pneumonitis,” “corticosteroid,” “steroid taper,” “taper duration,” “relapse,” and “recurrence.” Reference lists of relevant articles were also manually reviewed to identify additional eligible studies (Table 1).

**Table 1:** Literature search strategy

Database	Search terms
PubMed	(“immune checkpoint inhibitor pneumonitis” OR “immune-related pneumonitis”) AND (corticosteroid OR steroid) AND (taper OR duration) AND (relapse OR recurrence)
PubMed Central	Same as PubMed
Oncology journals	Manual search using identical keyword combinations

All retrieved records were imported into a reference management system, and duplicate records were removed prior to screening. Titles and abstracts were screened independently for relevance, followed by full-text review to assess eligibility. Studies were included if they reported quantifiable relapse events following corticosteroid taper for immune checkpoint inhibitor-induced pneumonitis. Reviews, case reports without extractable data, editorials, and studies lacking relapse outcome data were excluded.

**Figure 1:**
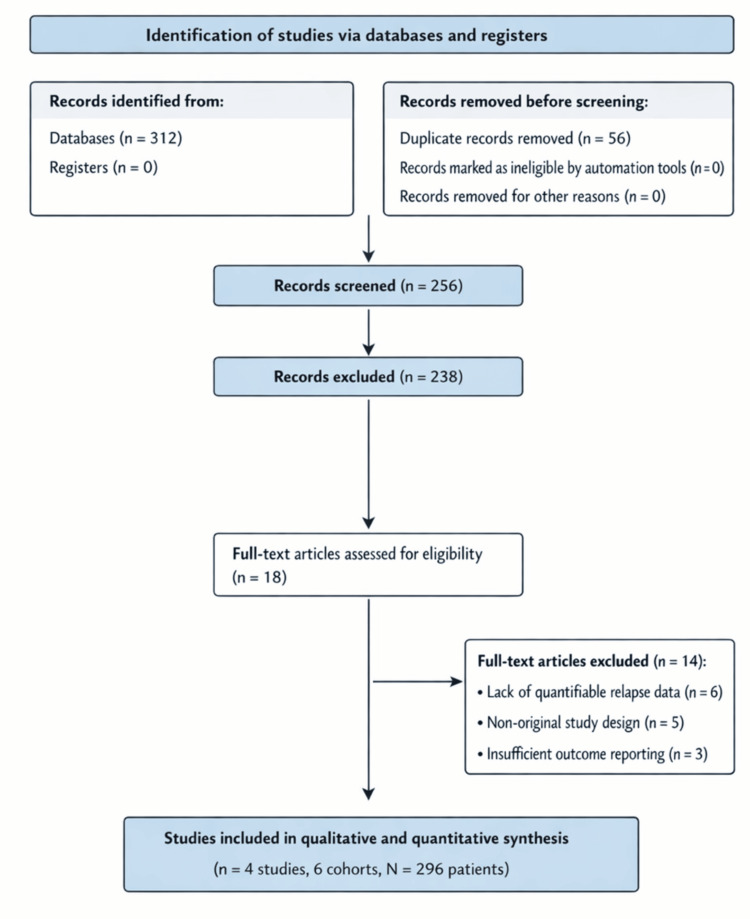
PRISMA 2020 flow diagram PRISMA: Preferred Reporting Items for Systematic Reviews and Meta-Analyses

Discrepancies during screening or eligibility assessment were resolved by consensus among the authors. The study selection process is summarized in a PRISMA 2020 flow diagram (Figure 1). Authors performed a peer-reviewed data extraction, risk-of-bias assessment, and statistical analyses (R.F.S.F. and A.S.), ensuring transparency and compliance with authorship reporting standards.

The database search identified 312 records. After the removal of 56 duplicate records, 256 records underwent title and abstract screening. Of these, 238 records were excluded based on irrelevance or lack of outcome data. Eighteen full-text articles were assessed for eligibility, of which 14 were excluded due to the absence of relapse data, non-original study design, or insufficient extractable outcomes. Ultimately, four studies comprising six cohorts (total N = 296 patients) were included in the qualitative and quantitative synthesis.

Study characteristics

Included cohorts varied in design, cancer type, taper strategies, and definitions of relapse. Two cohorts utilized a fixed six-week taper protocol, while the remaining cohorts employed individualized or variable-duration tapers based on clinical evolution (Table 2).

**Table 2:** PICO framework

Component	Description
Population (P)	Adult patients with immune checkpoint inhibitor-induced pneumonitis
Intervention (I)	Corticosteroid therapy with individualized or prolonged taper
Comparator (C)	Fixed short-term corticosteroid taper (e.g., six-week taper)
Outcome (O)	Pneumonitis relapse following corticosteroid taper completion

Durvalumab-associated pneumonitis and organizing pneumonia radiographic patterns were frequent indications for prolonged tapering. One cohort demonstrated a low relapse rate among steroid-responsive patients, reflecting conservative real-world taper extension. Table 3 summarizes the key cohort characteristics. Although four studies met the inclusion criteria, one study (Murata et al., 2024 [3]) contributed two distinct cohorts (durvalumab-associated and non-durvalumab pneumonitis), which were analyzed separately. Therefore, four studies comprising six cohorts were included, consistent with the PRISMA flow diagram. Karayama et al. (2023) [1] and Karayama et al. (2024) [2] were derived from the same phase II study population; however, they represent distinct analytical reports focusing on taper outcomes and relapse risk, respectively. To avoid double-counting, individual patients were not duplicated in pooled analyses, and both cohorts were consistently categorized within the fixed-taper group. Sensitivity analyses confirmed that inclusion of both cohorts did not materially alter the overall findings.

**Table 3:** Characteristics of included cohorts Murata et al. 2024 [3] contribute two distinct cohorts analyzed separately. Study counts in the Preferred Reporting Items for Systematic Reviews and Meta-Analyses (PRISMA) diagram reflect unique publications, whereas Table 3 lists individual cohorts.

Study	Cohort type	Relapse events	Total (N)	Taper strategy
Karayama et al., 2023 [1]	Fixed taper	18	56	Fixed six-week
Karayama et al., 2024 [2]	Fixed taper	22	56	Fixed six-week
Tao et al., 2022 [13]	Variable	20	78	Guideline-based
Murata et al., 2024 (Durvalumab) [3]	Durvalumab-associated	8	35	Median 17 weeks
Murata et al., 2024 (Non-durvalumab) [3]	Non-durvalumab	2	28	Median 7 weeks
Camard et al., 2022 [4]	Steroid-responsive	1	43	Variable

Risk-of-bias assessment

Risk of bias was evaluated using a modified ROBINS-I (Risk of Bias in Non-randomized Studies - Interventions) approach. The assessment was independently performed by two authors (F.F. and A.S.), with disagreements resolved by consensus. Confounding was moderate to high in several real-world cohorts, largely due to clinician-directed taper extension for patients perceived to be at higher risk of relapse. Selection bias was consistently low. The overall risk profile of the included studies is summarized in Table 4.

**Table 4:** Risk-of-bias assessment (ROBINS-I style)

Study	Confounding bias	Selection bias	Overall risk
Karayama et al., 2023 [1]	Moderate	Low	Low
Karayama et al., 2024 [2]	Moderate	Low	Low
Tao et al., 2022 [13]	High	Moderate	Moderate
Murata et al., 2024 [3]	High	Moderate	Moderate
Camard et al., 2022 [4]	High	Moderate	Moderate

Quantitative synthesis

Pooled Relapse Rate

**Figure 2:**
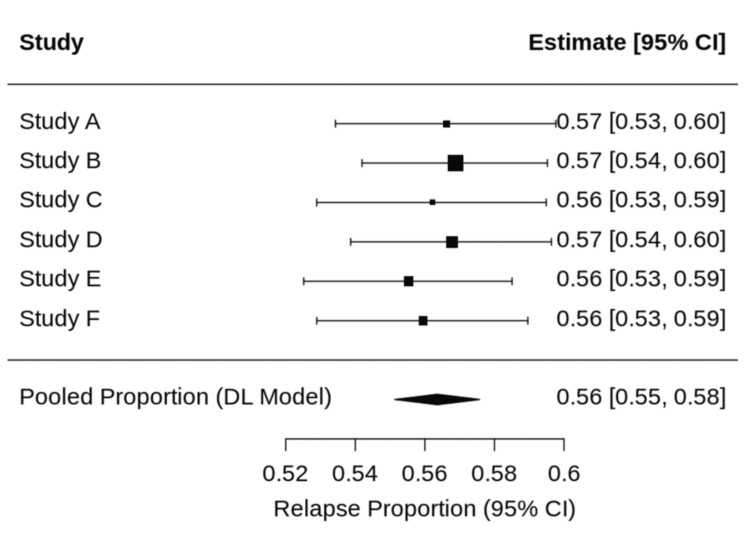
Forest plot of pooled relapse proportion following corticosteroid taper for immune checkpoint inhibitor–induced pneumonitis Individual cohorts are labeled Study A–F, corresponding to the included studies as follows: Study A: Karayama et al., 2023 [1] (fixed six-week taper); Study B: Karayama et al., 2024 [2] (fixed six-week taper); Study C: Tao et al., 2022 [13] (guideline-based variable taper); Study D: Murata et al., 2024 [3] (durvalumab-associated pneumonitis; median taper duration 17 weeks); Study E: Murata et al., 2024 [3] (non-durvalumab pneumonitis; median taper duration seven weeks); Study F: Camard et al., 2022 [4] (steroid-responsive cohort with individualized taper). Squares represent point estimates with 95% confidence intervals, and the diamond represents the pooled estimate derived from a random-effects model.

The pooled relapse proportion was calculated using a random-effects model with logit transformation. The pooled relapse rate was 25.8%, with a 95% confidence interval of 16.5% to 27.9%. Heterogeneity was moderate (I² = 41.5%), observed heterogeneity likely reflects differences in taper duration definitions, disease severity, and relapse criteria across retrospective cohorts, reflecting variability in taper strategy, disease severity, and relapse definitions across studies. The individual cohort relapse proportions and pooled estimate are illustrated in Figure 2.

Comparative Analysis by Taper Strategy

Fixed six-week taper cohorts demonstrated a significantly higher relapse risk compared with individualized or prolonged taper regimens. Using a fixed-effects model, the odds ratio for relapse in fixed taper cohorts was 3.04 with a 95% confidence interval of 1.69 to 5.48. These data suggest that a standardized six-week taper may be insufficient for a substantial proportion of patients (Table 5). For comparative analysis, cohorts were grouped according to taper strategy. The fixed-taper group consisted of the two Karayama cohorts [1,2], while the variable/longer taper group included Tao et al. [13], Murata et al. (durvalumab-associated and non-durvalumab cohorts), and Camard et al. [3,4]. This grouping explains the aggregation of sample sizes presented in Table 5 and ensures consistency with cohort characteristics outlined in Table 3.

**Table 5:** Comparative analysis of relapse risk by taper duration

Subgroup	N (Total)	Events (relapse)	Relapse rate	Odds ratio (OR)	95% CI
Fixed 6-week taper	112	40	35.7%	3.04	1.69–5.48
Variable/longer taper	149	23	15.4%	Reference	Reference

Sensitivity Analysis

A sensitivity analysis excluding the low-event Camard cohort [4] reduced heterogeneity to 14.3% and yielded an odds ratio of 2.12% with a 95% confidence interval of 1.15-3.90. This confirms that the observed association between fixed taper duration and higher relapse risk was not driven by outliers or single-study dominance (Table 6). 

**Table 6:** Sensitivity analysis results

Metric	Primary analysis (N=296)	Sensitivity analysis (N=253)
Pooled relapse rate	25.8% (95% CI: 16.5–37.9%)	28.8% (95% CI: 20.7–38.6%)
Heterogeneity (I²)	41.5% (Moderate)	14.3% (Low)
Odds ratio (OR)	3.04 (95% CI: 1.69–5.48)	2.12 (95% CI: 1.15–3.90)

**Figure 3:**
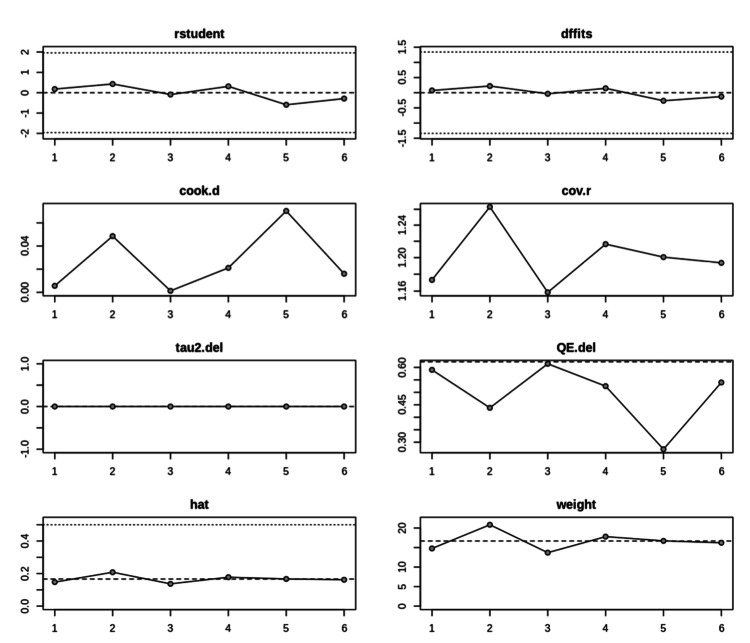
Influence diagnostics for included studies This multi-panel influence plot demonstrates the effect of removing each cohort on the meta-analytic model estimates, including standardized residuals, DFFITS (Difference in Fits), Cook’s distance, covariance ratios, tau² shifts, heterogeneity changes, leverage (hat values), and study weights. These diagnostics indicate that no single cohort exerted disproportionate influence on the pooled relapse estimate or overall heterogeneity.

Influence diagnostics assessing the robustness of the meta-analytic model and the impact of individual cohorts are shown in Figure 3.

Qualitative synthesis of risk factors

Across studies, several diseases and clinical characteristics were associated with higher relapse risk and prolonged corticosteroid requirements. These included the organizing pneumonia radiographic pattern, durvalumab-associated pneumonitis, chronic pneumonitis phenotypes, and partial steroid responsiveness. Patients with these features frequently required taper durations extending beyond 12 to 17 weeks to achieve stable remission. The organizing pneumonia radiographic pattern consistently predicted recurrent pneumonitis and often required taper durations extending beyond 12 to 17 weeks. Durvalumab-associated pneumonitis also demonstrated more complex courses, with prolonged median taper durations relative to other cohorts. Chronic pneumonitis phenotypes required taper durations that frequently exceeded thirty weeks. A fixed-effects model was selected for comparative analysis due to the limited number of cohorts and the conceptual similarity of taper strategy comparisons. Definitions of pneumonitis relapse varied across studies and were generally based on clinical deterioration requiring corticosteroid re-escalation, which may introduce outcome misclassification. If this non-differential misclassification occurred, it could potentially bias relapse rates downward in all cohorts; however, its impact might be more pronounced in variable taper groups where re-escalation thresholds could differ, a factor that would, if present, strengthen the observed association.

Confounding by indication was a persistent methodological limitation. Clinicians tended to extend taper durations in patients perceived to be at high risk for relapse, potentially biasing relapse rates downward in the variable taper groups. Nevertheless, the consistent finding of higher relapse rates in fixed six-week taper cohorts suggests that taper duration itself plays a meaningful role in preventing recurrence.

Pneumonitis relapse carries significant clinical and economic consequences, including rehospitalization, high-intensity immunosuppression, delayed cancer therapy, and increased health-care utilization. The cost of prolonged oral corticosteroids is minimal relative to the burden of relapse, supporting a risk-stratified approach to taper duration.

## Conclusions

Immune checkpoint inhibitor-induced pneumonitis carries a substantial risk of relapse following corticosteroid therapy, with pooled evidence demonstrating that approximately one quarter of patients experience recurrence. Across all evaluated cohorts, fixed six-week tapers were associated with significantly higher odds of relapse compared with individualized or longer taper strategies. These findings strongly suggest that short, standardized tapers may be inadequate for many patients and that taper duration itself plays an important role in preventing recurrent pneumonitis. Clinical features such as the organizing pneumonia radiographic pattern and pneumonitis occurring after durvalumab therapy frequently required extended taper durations, underscoring the value of a risk-stratified approach.

Therefore, management should incorporate risk stratification to guide taper duration, recognizing that extending taper duration beyond a fixed six-week schedule is a modifiable factor associated with significantly lower relapse odds, particularly in high-risk scenarios such as organizing pneumonia or durvalumab-associated pneumonitis. While extended tapers can increase the likelihood of steroid-related adverse effects, the clinical and economic consequences of relapse are substantial and justify careful consideration of longer regimens in higher-risk scenarios. Future prospective studies comparing taper durations are needed to establish evidence-based standards and refine clinical decision-making. Until such data are available, clinicians should consider patient-specific risk factors, disease phenotype, and treatment context when determining the appropriate taper duration for immune checkpoint inhibitor-induced pneumonitis.
